# The efficacy of niacin supplementation in type 2 diabetes patients: Study protocol of a randomized controlled trial

**DOI:** 10.1097/MD.0000000000022272

**Published:** 2021-03-26

**Authors:** Xiaoying Yan, Shunyu Wang

**Affiliations:** Department of General Practice, The Second Affiliated Hospital of Dalian Medical University, Liaoning, China.

**Keywords:** dyslipidemia, niacin, randomized controlled trial, study protocol, type 2 diabetic patients

## Abstract

**Background:**

Dyslipidemia is a main risk factor of cardiovascular disease in the diabetic patients. Niacin was found acutely to decrease the plasma concentration of free fatty acids by inhibiting their mobilization from adipose tissue. This present study is a double blinded, randomized, and prospective trial to determine the effect of niacin during dyslipidemia in type 2 diabetic patients.

**Methods:**

This randomized controlled, double-blinded, single center trial is carried out according to the principles of Declaration of Helsinki. This present study was approved in institutional review committee of the Second Affiliated Hospital of Dalian Medical University. All the patients received the informed consent. Diabetic patients were randomized (1:1) to receive 3-month treatment with extended-release niacin or matching placebo. The major outcome of our present study was the change in the level of HbA1c from the baseline to week 12. Secondary outcome measures contained the levels of fasting blood glucose, the concentrations of serum transaminase, the other laboratory variables, and self-reported adverse events. The *P* < .05 was regarded as statistically significant.

**Results:**

We assumed that adding the niacin to the medication in patients with type 2 diabetes would reduce dyslipidemia and achieve target lipid levels.

**Trial registration:**

This study protocol was registered in Research Registry (researchregistry5925).

## 1 Introduction

Dyslipidemia is a main risk factor of cardiovascular disease in the diabetic patients. Diabetic dyslipidemia is characterized by an increased low density lipoprotein (LDL) -cholesterol particles concentration, low concentration of high density lipoprotein (HDL) -cholesterol, and a high concentration of plasma triglyceride.^[1–5]^ The changes in lipid related to the diabetes are due to an increase in flux of free fatty acids secondary to the insulin resistance.^[6]^

Niacin was found acutely to decrease the plasma concentration of free fatty acids by inhibiting their mobilization from adipose tissue.^[7–9]^ The term “niacin” is generally defined as the nicotinic acid, but can also be more broadly defined as “nicotinic acid, nicotinamide, as well as the derivatives with the nicotinamide biological activity”. Whether conversion to the nicotinic acid or other compounds involving nicotinamide, nicotinic acid or their releasable parts can be considered “niacin” depends primarily on the interpretation of evidence for uptake and the metabolic rates, and the biological effects of the compound, and/or release of chemical components that generate the biological effects similar to those of major forms of the niacin.^[10–16]^ Extended release nicotinic acid is an effective lipid-modifying agents at a dose sufficient to generate the pharmacological activities and has a wide range of effects, containing the effects aimed at reducing the risks related to high LDL cholesterol, low HDL cholesterol, high triglyceridemia and high lipoprotein. Niacin offers novel opportunities for the patients to reach target levels of lipid.^[17–22]^

This present study is a double blinded, randomized, and prospective trial to determine the effect of niacin during dyslipidemia in type 2 diabetic patients. We assumed that adding the niacin to the medication in patients with type 2 diabetes would reduce dyslipidemia and achieve target lipid levels.

## 2 Material and method

### 2.1 Study design

This randomized controlled, double-blinded, single center trial is carried out according to the principles of Declaration of Helsinki. This present study was approved in institutional review committee of the Second Affiliated Hospital of Dalian Medical University (DL0094130). All the patients received the informed consent. It was also registered at the Research Registry (researchregistry5925).

### 2.2 Eligibility criteria

Inclusion criteria included:

1. type 2 diabetic patients;
2. aged between 30 and 65 years old;
3. the statin therapy with stable-dose for six weeks or more;
4. serum low-density lipoprotein-cholesterol ≤2.5 mmol/L and endothelial dysfunction.

Exclusion criteria included: Patients with poor control of diabetes mellitus (HbA1c .9.0%), renal disease, peptic ulcer disease, liver disease, and parents with the history of diabetic coma or ketoacidosis, hyperuricemia, and gout.

### 2.3 Randomization and blinding

**Figure 1:**
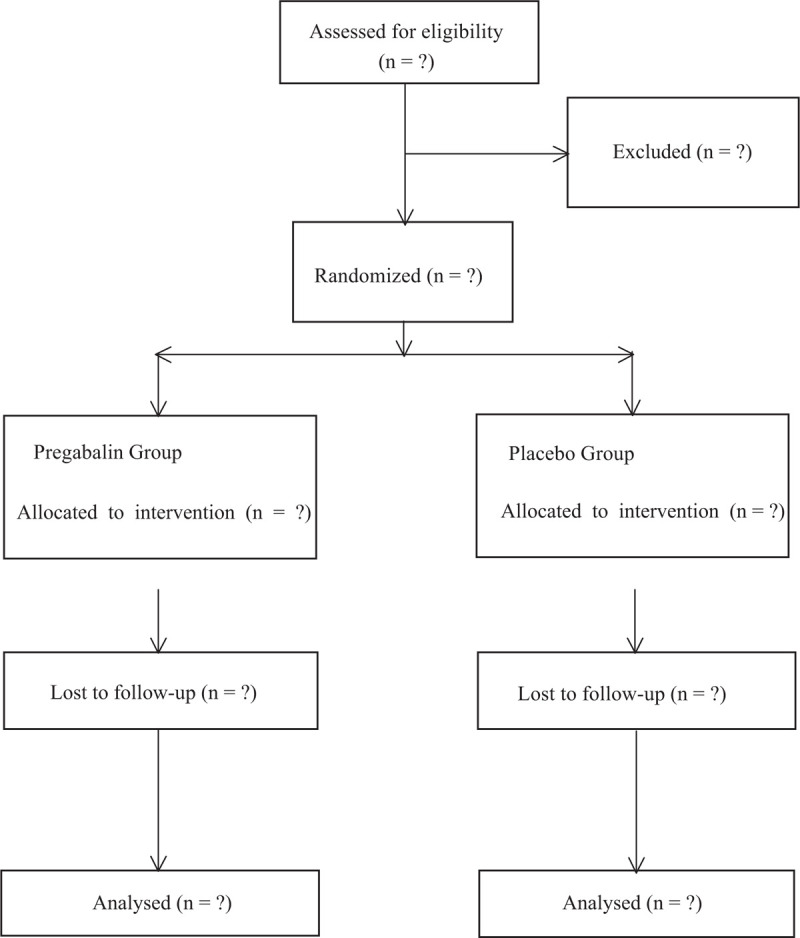
Flow diagram of the study.

The number of envelopes in each research group was equal and they were produced via a research assistant through utilizing a computer-based random number generator who did not participate in any follow-up studies or contact with other members of study team throughout the whole study period. He prepared 80 identical, sealed bound, opaque, sequentially numbered envelopes; forty envelopes containing the instructions for group A mixing solution and other forty envelopes for the group B. These envelopes were placed in file with the principal investigator (Fig. 1).

### 2.4 Intervention and control groups

Diabetic patients were then randomized (1:1) to receive 3-month treatment with extended-release niacin or matching placebo. In randomized trials, the dose of niacin or its placebo was enhanced to 750 mg twice a day, then to 1000 mg, afterwards, increases to 1500 mg twice a day at intervals of six weeks, or until achieving the maximum tolerated dose. In order to prevent flushing, the subjects who did not take aspirin started taking aspirin 100 mg/day at least three weeks before the measurements of baseline vascular. During follow-up period, the levels of fasting glucose were monitored every six weeks. If the level of fasting blood glucose was greater than 10.5 mmol/L, the level of HbA1c needed to be measured, and if it was greater than 10.0%, we should reduce the dose of niacin (or its placebo). There was no change in the medication of diabetic patients during the treatment period.

### 2.5 Outcome measures

The major outcome of our present study was the change in the level of HbA1c from the baseline to week 12. Secondary outcome measures contained the levels of fasting blood glucose, the concentrations of serum transaminase, the other laboratory variables, and self-reported adverse events. The other laboratory variables involved the apolipoprotein, lipoprotein, and plasma lipid, uric acid, glucose, as well as the insulin concentrations. The serum levels of uric acid, glucose and the serum transaminase concentrations or the plasma were determined in local clinical laboratory via a standard automatic analyzer.

### 2.6 Statistical analysis

Statistical analysis was conducted through utilizing the Statistical Package for Social Sciences (SPSS for Windows, release 12.0; SPSS Inc, Chicago, IL). The statistical analysis was carried out by independent experts who did not participate in the research program. The values of mean (range) and median were presented. Non-paired *t* test was utilized for the numerical data of normal distribution. Non-parametric simulation was utilized where appropriate. The comparison of categorical variables was performed by χ^2^ test. The *P* < .05 was regarded as statistically significant.

## 3 Discussion

Despite progress in prevention and treatment of the cardiovascular diseases, incidence rate and mortality rate of diabetic patients are still strikingly high, second only to the cardiovascular diseases. Nicotinic acid (also know as niacin), is an indispensable B-complex vitamin. At the pharmacological dose, it is an effective drug to reduce plasma triglyceride and increase HDL-cholesterol, and has moderate activities on the LDL-cholesterol. Niacin has been proven to reverse the coronary atherosclerosis and decrease the coronary mortality rate. Niacin possesses complex actions mechanisms, which has not been completely elucidated. This present study is a double blinded, randomized, and prospective trial to determine the effect of niacin during dyslipidemia in type 2 diabetic patients. We assumed that adding the niacin to the medication in patients with type 2 diabetes would reduce dyslipidemia and achieve target lipid levels.

## Author contributions

**Conceptualization:** Shunyu Wang.

**Data curation:** Shunyu Wang.

**Formal analysis:** Xiaoying Yan, Shunyu Wang.

**Funding acquisition:** Shunyu Wang.

**Investigation:** Xiaoying Yan.

**Methodology:** Shunyu Wang.

**Project administration:** Shunyu Wang.

**Resources:** Shunyu Wang.

**Software:** Xiaoying Yan.

**Supervision:** Shunyu Wang.

**Validation:** Xiaoying Yan.

**Writing – original draft:** Xiaoying Yan.

**Writing – review & editing:** Shunyu Wang.

## References

[1] Elam MB Hunninghake DB Davis KB. Effect of niacin on lipid and lipoprotein levels and glycemic control in patients with diabetes and peripheral arterial disease: the ADMIT study: a randomized trial. Arterial Disease Multiple Intervention Trial. JAMA 2000;284:1263–70. 10979113 10.1001/jama.284.10.1263

[2] Fisman EZ Motro M Tenenbaum A. Impaired fasting glucose concentrations in nondiabetic patients with ischemic heart disease: a marker for a worse prognosis. Am Heart J 2001;141:485–90. 11231448 10.1067/mhj.2001.113219

[3] Sattar N Preiss D Murray HM. Statins and risk of incident diabetes: a collaborative meta-analysis of randomised statin trials. Lancet 2010;375:735–42. 20167359 10.1016/S0140-6736(09)61965-6

[4] Sazonov V Maccubbin D Sisk CM. Effects of niacin on the incidence of new onset diabetes and cardiovascular events in patients with normoglycaemia and impaired fasting glucose. Int J Clin Pract 2013;67:297–302. 23521322 10.1111/ijcp.12089

[5] Guyton JR Fazio S Adewale AJ. Effect of extended-release niacin on new-onset diabetes among hyperlipidemic patients treated with ezetimibe/simvastatin in a randomized controlled trial. Diabetes Care 2012;35:857–60. 22338103 10.2337/dc11-1369 PMC3308290

[6] Rajanna V Campbell KB Leimberger J. Elevation of fasting morning glucose relative to hemoglobin A1c in normoglycemic patients treated with niacin and with statins. J Clin Lipidol 2012;6:168–73. 22385550 10.1016/j.jacl.2011.12.008

[7] Goldberg RB Bittner VA Dunbar RL. Effects of extended-release niacin added to simvastatin/ezetimibe on glucose and insulin values in AIM-HIGH. Am J Med 2016;129: 753.e13–22. 10.1016/j.amjmed.2016.02.039 PMC4914402 27036394

[8] Brown BG Zhao XQ Chait A. Simvastatin and niacin, antioxidant vitamins, or the combination for the prevention of coronary disease. N Engl J Med 2001;345:1583–92. 11757504 10.1056/NEJMoa011090

[9] Whitney EJ Krasuski RA Personius BE. A randomized trial of a strategy for increasing high-density lipoprotein cholesterol levels: effects on progression of coronary heart disease and clinical events. Ann Intern Med 2005;142:95–104. 15657157 10.7326/0003-4819-142-2-200501180-00008

[10] Bays HE Ose L Fraser N. A multicenter, randomized, double-blind, placebo-controlled, factorial design study to evaluate the lipid-altering efficacy and safety profile of the ezetimibe/simvastatin tablet compared with ezetimibe and simvastatin monotherapy in patients with primary hypercholesterolemia. Clin Ther 2004;26:1758–73. 15639688 10.1016/j.clinthera.2004.11.016

[11] Robinson JG Ballantyne CM Grundy SM. Lipid-altering efficacy and safety of ezetimibe/simvastatin versus atorvastatin in patients with hypercholesterolemia and the metabolic syndrome (from the VYMET study). Am J Cardiol 2009;103:1694–702. 19539078 10.1016/j.amjcard.2009.05.003

[12] Simons L Tonkon M Masana L. Effects of ezetimibe added to on-going statin therapy on the lipid profile of hypercholesterolemic patients with diabetes mellitus or metabolic syndrome. Curr Med Res Opin 2004;20:1437–45. 15383192 10.1185/030079904x2321

[13] Sattar N Gaw A Scherbakova O. Metabolic syndrome with and without C-reactive protein as a predictor of coronary heart disease and diabetes in the West of Scotland Coronary Prevention Study. Circulation 2003;108:414–9. 12860911 10.1161/01.CIR.0000080897.52664.94

[14] Howard BV Roman MJ Devereux RB. Effect of lower targets for blood pressure and LDL cholesterol on atherosclerosis in diabetes—the SANDS randomized trial. JAMA 2008;299:1678–89. 18398080 10.1001/jama.299.14.1678 PMC4243925

[15] Avellone G Di Garbo V Guarnotta V. Efficacy and safety of long-term ezetimibe/simvastatin treatment in patients with familial hypercholesterolemia. Int Angiol 2010;29:514–24. 21173733

[16] Anderson RA Evans ML Ellis GR. The relationships between post-prandial lipaemia, endothelial function and oxidative stress in healthy individuals and patients with type 2 diabetes. Atherosclerosis 2001;154:475–83. 11166782 10.1016/s0021-9150(00)00499-8

[17] Lee JMS Robson MD Yu LM. Effects of high-dose modified-release nicotinic acid on atherosclerosis and vascular function: a randomised, placebo-controlled, magnetic resonance imaging study. J Am Coll Cardiol 2009;54:1787–94. 19874992 10.1016/j.jacc.2009.06.036

[18] Grey E Bratteli C Glasser SP. Reduced small artery but not large artery elasticity is an independent risk marker for cardiovascular events. Am J Hypertens 2003;16:265–9. 12670741 10.1016/s0895-7061(02)03271-5

[19] Elam MB Hunninghake DB Davis KB. Effect of niacin on lipid and lipoprotein levels and glycemic control in patients with diabetes and peripheral arterial disease. JAMA 2000;284:1263–70. 10979113 10.1001/jama.284.10.1263

[20] McKenney J Bays H Koren M. Safety of extended-release niacin/laropiprant in patients with dyslipidemia. J Clin Lipidol 2010;4:105–12. 21122637 10.1016/j.jacl.2010.02.002

[21] Pang J Chan DC Hamilton SJ. Effect of niacin on high-density lipoprotein apolipoprotein A-I kinetics in statin-treated patients with type 2 diabetes mellitus. Arterioscler Thromb Vasc Biol 2014;34:427–32. 24285582 10.1161/ATVBAHA.113.302019

[22] Zhang LH Kamanna VS Ganji SH. Niacin increases HDL biogenesis by enhancing DR4-dependent transcription of ABCA1 and lipidation of apolipoprotein A-I in HepG2 cells. J Lipid Res 2012;53:941–50. 22389325 10.1194/jlr.M020917 PMC3329393

